# Finite Element Analysis of the Flexural Performance of ECC–Concrete Composite Beams Reinforced with GFRP–Steel Composite Bars

**DOI:** 10.3390/polym18172157

**Published:** 2026-09-03

**Authors:** Yu Ling, Xin Luo, Shuo Xu, Zile Feng, Junzhe Qin, Yicong Zhong, Yongjian Cai

**Affiliations:** 1 Guangzhou Power Supply Bureau, Guangdong Power Grid Co., Ltd., Guangzhou 510665, China; 2 School of Civil and Transportation Engineering, Guangdong University of Technology, Guangzhou 510006, China; 3 School of Civil and Transportation, Foshan University, Foshan 528225, China; 4 School of Intelligent Transportation and Engineering, Guangzhou Maritime University, Guangzhou 510725, China

**Keywords:** steel–FRP composite bar, engineered cementitious composite, ECC–concrete composite beam, flexural behavior, finite element analysis

## Abstract

GFRP-reinforced concrete structures often suffer from insufficient ductility and limited crack control capability. This study investigates the flexural behavior of ECC–concrete composite beams reinforced with steel–FRP composite bars (SFCBs) using finite element analysis. A three-dimensional nonlinear finite element model was developed in ABAQUS and validated against four-point bending tests of eight composite beam specimens. The model incorporated a bilinear constitutive relationship for SFCBs and a cohesive interface model to simulate the interaction between ECC and concrete. Based on the validated model, the effects of reinforcement type and ECC replacement height on the flexural performance of composite beams were evaluated. The results showed that the proposed model accurately reproduced the load–deflection responses, strain development, and failure processes of the tested beams. The reinforcement type significantly affected the overall structural behavior, resulting in different load-carrying characteristics and deformation responses. Increasing the ECC replacement height mainly improved the cracking resistance by increasing the cracking load and promoting a more uniform crack distribution, while its effects on the yield and ultimate loads were limited. ECC primarily contributed to crack control and damage mitigation, whereas SFCBs influenced the overall load-carrying behavior of the composite beams. These findings provide insights into the design and performance evaluation of ECC–concrete composite beams reinforced with SFCBs.

## 1. Introduction

Steel-reinforcement corrosion in coastal, marine, and deicing salt environments remains a critical issue affecting the durability and service life of concrete structures. Fiber-reinforced polymer (FRP) bars have been recognized as promising alternatives to conventional steel reinforcement due to their excellent corrosion resistance, high specific strength, and electromagnetic neutrality [1,2,3]. However, commonly used glass fiber-reinforced polymer (GFRP) bars generally exhibit a relatively low elastic modulus and linear-elastic behavior until brittle failure, which often results in excessive deflection, poor crack width control, and insufficient ductility in FRP-reinforced concrete beams under flexural loading [4,5]. These limitations have hindered the wider application of FRP reinforcement in flexural members.

To achieve a balance between durability and ductility, two approaches have been proposed. One approach is the use of hybrid reinforcement systems combining steel bars and FRP bars, in which the plastic deformation capacity of steel after yielding can improve the ductility of structural members [6,7,8]. The other approach is the development of steel-FRP composite bars (SFCBs), which integrate the ductility of steel and the corrosion resistance of FRP into a single reinforcing element [9,10,11,12]. Qu et al. [13] conducted an early investigation on the flexural behavior of concrete beams reinforced with a combination of GFRP bars and steel reinforcement, demonstrating that hybrid reinforcement could effectively enhance the ductility of FRP-reinforced beams. Subsequently, Xiao et al. [14] performed flexural tests on concrete beams reinforced with GFRP–steel composite bars and reported that these members exhibited a distinct post-yield stiffness and improved ductile behavior, indicating the potential of composite reinforcement for enhancing flexural performance.

In addition to the reinforcement system, the tensile properties of the cementitious matrix also play an important role in controlling crack propagation and deformation capacity of FRP- or composite-reinforced flexural members [15,16,17,18,19]. Engineered cementitious composite (ECC) is a high-ductility cementitious material designed according to micromechanical principles and characterized by strain-hardening behavior and multiple cracking [20,21,22,23,24]. ECC can achieve tensile strain capacities of 2–8% [25,26], while maintaining crack widths generally below 60 μm [27,28]. By incorporating ECC in the tensile region of beams, its superior crack-control capability and energy dissipation capacity can be utilized without substantially increasing material costs. Zhang et al. [29] reported that layered ECC–concrete composite beams exhibited improved crack dispersion and deformation performance under flexural loading. Ge et al. [30] further demonstrated that introducing an ECC layer in the tensile zone of FRP-reinforced concrete beams could effectively enhance stiffness and cracking resistance.

Recently, increasing attention has been paid to the synergistic interaction between ECC–concrete composite beams and hybrid reinforcement systems [31]. Ge et al. [32] experimentally investigated ECC–concrete composite beams reinforced with hybrid FRP bars and steel reinforcement, revealing that the combination of an ECC layer and hybrid reinforcement could improve the flexural capacity, yield moment, and overall stiffness of the beams. Yuan and Hu [33] further compared the flexural behavior of ECC beams and ECC–concrete composite beams reinforced with hybrid FRP/steel reinforcement, and found that ECC could alleviate crack localization caused by the brittle tensile behavior of conventional concrete. More recently, Bai et al. [34] applied SFCBs in ECC–concrete composite beams and found that the interaction between ECC and composite reinforcement improved crack development, load-carrying capacity, and ductility. Experimental investigations by Wang et al. [35] on GFRP-reinforced ECC–concrete composite beams also demonstrated the beneficial effects of ECC on flexural capacity, ductility, and crack control. Meanwhile, Shbeeb et al. [36] developed a nonlinear finite element model for ECC–concrete composite beams reinforced with hybrid FRP/steel bars, indicating that numerical methods can provide an effective approach for understanding the mechanical behavior and conducting parametric investigations of such composite members. In addition to conventional finite element modelling, data-driven methods have recently been applied to response prediction and damage assessment of FRP-related structural systems. Khatir et al. [37,38] demonstrated the applicability of machine-learning-based approaches to strain prediction in FRP-strengthened reinforced concrete beams and damage prediction in FRP-composite structural components.

Although previous studies have demonstrated the beneficial synergistic effects of ECC and FRP/steel or SFCB reinforcement in flexural members, investigations on ECC–concrete composite beams reinforced with SFCB remain limited. In particular, the interaction among composite reinforcement, ECC layers, and normal concrete under different ECC replacement heights, as well as the corresponding crack propagation characteristics and stiffness evolution, require further investigation. Moreover, three-dimensional nonlinear finite element models with experimental validation for such composite beams are still relatively scarce [32,35,36]. Previous numerical studies have mainly focused either on ECC–concrete composite beams with conventional or hybrid reinforcement or on SFCB-reinforced concrete beams without an ECC–concrete interface. Therefore, the present study develops a three-dimensional nonlinear FE model for SFCB-reinforced ECC–concrete composite beams, in which the ECC–concrete interface is explicitly represented using a surface-based cohesive interaction rather than a perfectly bonded assumption. The validated model is further used to investigate the effects of ECC replacement height and SFCB reinforcement characteristics on the flexural response of the composite beams.

## 2. Finite Element Analysis

### 2.1. Overview of Finite Element Model

**Figure 1:**
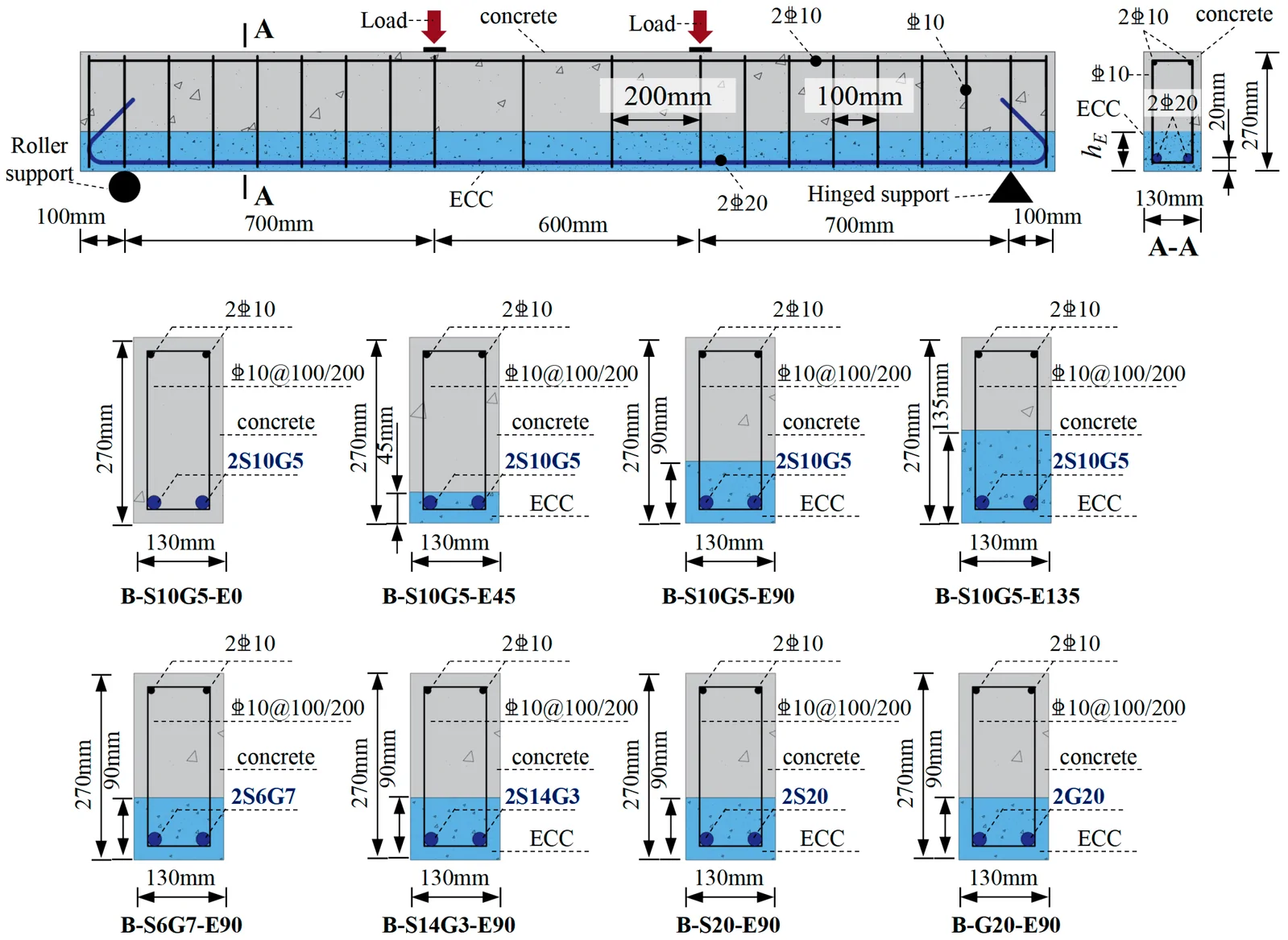
Geometric information of beam specimens.

**Figure 2:**
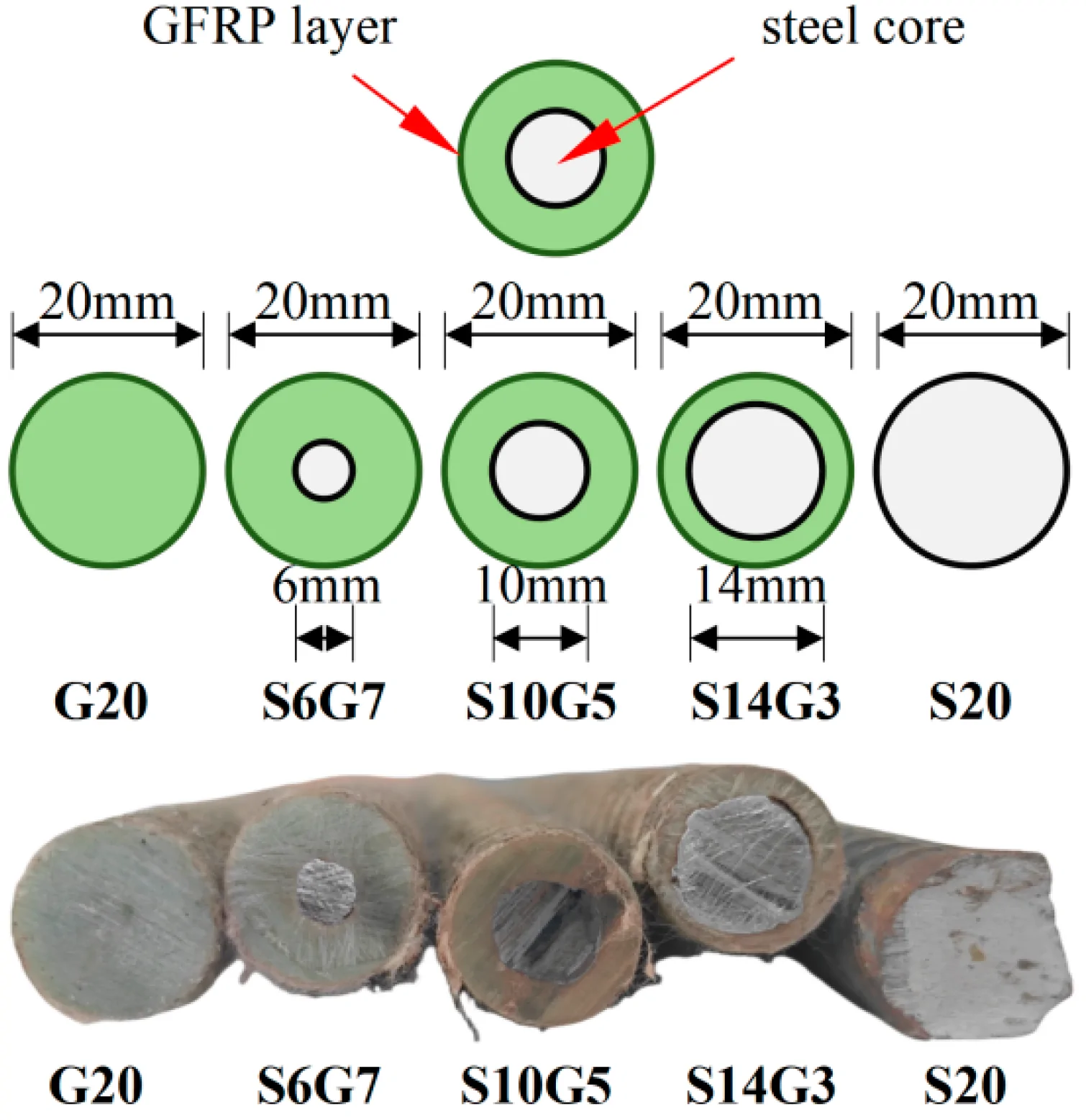
Diagram of Reinforcement.

To simulate the overall flexural behavior of SFCB-reinforced ECC–concrete composite beams under four-point bending, a three-dimensional nonlinear finite element model was established using ABAQUS 2016. No custom source code or independently developed computational program was used in the numerical simulations. The finite element models corresponded to the eight composite beam specimens tested previously [39], as shown in Figure 1. The specimens had a cross-sectional dimension of 130 mm × 270 mm, an overall length of 2200 mm, a clear span of 2000 mm, and a pure bending region of 600 mm. The main parameters of the specimens included the ECC replacement height in the tensile zone and the steel ratio of the bottom longitudinal reinforcement. The ECC replacement heights were set as 0, 45, 90, and 135 mm. For the specimens with an ECC replacement height of 90 mm, the bottom longitudinal reinforcement consisted of 20 mm diameter GFRP bars, SFCBs with different steel ratios, and conventional steel bars, as shown in Figure 2. In this study, G20 and S20 denote the specimens reinforced with pure GFRP bars and pure steel bars, respectively. S10G5, S14G3, and S6G7 represent the SFCBs with core steel-bar diameters of 10 mm, 14 mm, and 6 mm, respectively. The geometric dimensions, reinforcement configurations, and loading conditions in the finite element models were consistent with those used in the experimental program.

### 2.2. Constitutive Models

#### 2.2.1. Concrete

The Concrete Damaged Plasticity (CDP) model available in ABAQUS was adopted to simulate the behavior of normal concrete [40]. This model can capture the nonlinear response of concrete under both tensile cracking and compressive crushing, making it suitable for analyzing the entire flexural process of reinforced concrete beams. According to the preliminary experimental results [39], the average cubic compressive strength of normal concrete was 46.72 MPa, the elastic modulus was 33.85 GPa, and the Poisson’s ratio was set as 0.22. As expressed in Equations (1)–(6), the stress–strain relationships of concrete under compression and tension were determined according to the Code for Design of Concrete Structures (GB 50010-2010) [41]. These relationships were further converted into the inelastic strain and damage parameters required by ABAQUS for the implementation of the CDP model.

The stress–strain relationship of concrete under compression is given as:

(1) $$\sigma =\left(1-d_{c}\right)E_{c}\varepsilon$$

(2) $$d_{c}=\left\{\begin{array}{cc} 1-\frac{\rho_{c}n}{n-1+x^{n}} & ,x\leq 1\\ 1-\frac{\rho_{c}}{\alpha_{c}{\left(x-1\right)}^{2}+x} & ,x>1 \end{array}\right.$$

(3) $$\rho_{c}=\frac{f_{c}}{E_{c}\varepsilon_{c}},\;n=\frac{E_{c}\varepsilon }{E_{c}\varepsilon_{c}-f_{c}}\;\mathrm{and}\;x=\frac{\varepsilon }{\varepsilon_{c}}$$

where *α_c_*, *f_c_*, *ε_c_* and *d_c_* represent the shape parameters of the descending branch, compressive strength, peak compressive strain, and damage evolution parameter. The stress–strain relationship of concrete under tension is given as:

(4) $$\sigma =\left(1-d_{t}\right)E_{c}\varepsilon$$

(5) $$d_{t}=\left\{\begin{array}{cc} 1-\rho_{t}\left(1.2-0.2x^{5}\right) & ,x\leq 1\\ 1-\frac{\rho_{t}}{\alpha_{t}{\left(x-1\right)}^{1.7}+x} & ,x>1 \end{array}\right.$$

(6) $$\rho_{t}=\frac{f_{t}}{E_{c}\varepsilon_{t}}\;\mathrm{and}\;x=\frac{\varepsilon }{\varepsilon_{t}}$$

where *α_t_*, *f_t_*, *ε_t_* and *d_t_* denote the shape parameters of the descending branch, tensile strength, peak tensile strain, and damage evolution parameter.

The compressive and tensile stress–strain relationships obtained from GB 50010-2010 were initially expressed in terms of nominal stress and strain and were converted into true stress and true strain for implementation in ABAQUS. The compressive inelastic strain (*ε_in_*) and tensile cracking strain (*ε_cr_*) required by the CDP model were calculated as:

(7) $$\varepsilon_{in}=\varepsilon_{c}-\frac{\sigma_{c}}{E_{0}}$$

(8) $$\varepsilon_{cr}=\varepsilon_{t}-\frac{\sigma_{t}}{E_{0}}$$

where *ε_c_* and *ε_t_* are the total strains under compression and tension, respectively, *σ_c_* and *σ_t_* are the stresses under compression and tension, respectively, and *E*_0_ is the initial elastic modulus. Based on the energy-equivalence principle proposed by Sidoroff [42], the compressive and tensile damage variables were calculated as:

(9) $$D_{c}=1-\sqrt{\frac{\sigma_{c}}{E_{0}\varepsilon_{c}}}$$

(10) $$D_{t}=1-\sqrt{\frac{\sigma_{t}}{E_{0}\varepsilon_{t}}}$$

The remaining CDP parameters were adopted from previous numerical studies without additional calibration against the present beam tests. Based on relevant study [43], the recommended damage plasticity parameters used for concrete in the FE model are listed in Table 1.

#### 2.2.2. ECC

The CDP model was adopted to describe the mechanical behavior of ECC. The tensile response of ECC was represented by a bilinear stress–strain relationship consisting of an elastic stage before first cracking and a strain-hardening stage after cracking. The characteristic parameters of the bilinear relationship were determined from the uniaxial tensile tests [39]. The first-cracking stress and strain were 2.89 MPa and 0.0004, respectively, while the peak tensile stress and corresponding strain were 8.04 MPa and 0.055. The initial elastic modulus of ECC was calculated from the ratio of the first-cracking stress to the corresponding strain.

The tensile damage variables required by the CDP model were determined with reference to the uniaxial tensile damage constitutive model for ECC proposed by Li et al. [44], in which the tensile damage evolution is related to the degradation of the secant stiffness after cracking. It should be noted that the present continuum CDP model represents the macroscopic tensile strain-hardening and damage response of ECC but does not explicitly reproduce the formation and propagation of individual multiple cracks. The post-peak tensile softening behavior was not considered because the peak tensile strain of ECC reached 0.055, and the compression-zone concrete reached its crushing strain before the ECC reached its peak tensile strain in all simulated specimens. Since the ECC layer was used only in the tensile region, its compressive properties were not experimentally characterized in the present study. For numerical implementation of the CDP model, the compressive strength and compressive stress–strain relationship of ECC were simplified to be the same as those adopted for normal concrete.

#### 2.2.3. Reinforcement

The conventional steel bars were modeled using an ideal elastic-plastic constitutive model. The elastic modulus and yield strength were 174.61 GPa and 439.6 MPa, respectively. The GFRP bars were simulated using a linear elastic constitutive model up to failure, with an elastic modulus and tensile strength of 46.48 GPa and 959.77 MPa, respectively. The tensile stress–strain relationship of SFCBs was calculated using the bilinear constitutive model, as expressed in Equations (11)–(13), to represent their mechanical response under tensile loading [45,46,47].

(11) $$\sigma =\left\{\begin{array}{cc} E_{I}\varepsilon & ,0<\varepsilon \leq \varepsilon_{y}\\ f_{y}+E_{II}\left(\varepsilon -\varepsilon_{y}\right) & ,\varepsilon_{y}<\varepsilon \leq \varepsilon_{u} \end{array}\right.$$

(12) $$E_{I}=\frac{E_{s}A_{s}+E_{f}A_{f}}{A}$$

(13) $$E_{II}=\frac{E_{f}A_{f}}{A}$$

where *ε_y_* is the yield strain of the steel core; *ε_u_* is the ultimate strain of FRP layer; *E_s_* and *A_s_* are the elastic modulus and cross-sectional area of the steel core; *E_f_* and *A_f_* are the elastic modulus and cross-sectional area of the FRP layer; and *A* is the total cross-sectional area of the SFCB. No obvious debonding or relative slip between the steel core and the GFRP layer was observed in the corresponding experiments. Therefore, the two components were assumed to remain well bonded and deform compatibly, and their combined tensile response was represented by the equivalent bilinear constitutive relationship adopted for the SFCB.

### 2.3. Simulation of the ECC–Concrete Interface

During the experimental investigation, it was observed that all composite beam specimens exhibited only localized horizontal slip or minor damage at the ECC–concrete interface near the main cracks under relatively high load levels, while no extensive interface debonding occurred. To accurately reproduce this behavior, a surface-based cohesive interaction available in ABAQUS was adopted to simulate the ECC–concrete interface.

The interface constitutive parameters were determined using the tensile mechanical model for ECC–concrete interfaces proposed by Tian et al. [48]. In the present study, the ECC layer was cast before the initial setting of the normal concrete, resulting in the interpenetration of fresh cement paste and aggregate interlocking at the interface. According to the classification proposed by Tian et al. [48], an interface roughness parameter of h = 0.85 and an average aggregate spacing of d = 5.5 mm were adopted. The cohesive interaction parameters calculated based on the Tian model [48] are summarized in Table 2.

### 2.4. Model Establishment

#### 2.4.1. Element Type and Mesh

The concrete and ECC layer were modeled using three-dimensional eight-node solid elements with reduced integration (C3D8R), while the longitudinal reinforcement and stirrups were simulated using two-node truss elements (T3D2). A mesh sensitivity analysis was conducted using specimen B-S10G5-E45. Since this specimen contains a relatively thin ECC layer, it was selected as a conservative case for evaluating the influence of ECC mesh discretization. Five mesh configurations, namely C40E25, C30E25, C25E25, C25E20, and C25E15, were examined, where CxEy denotes concrete and ECC element sizes of x mm and y mm, respectively.

**Figure 3:**
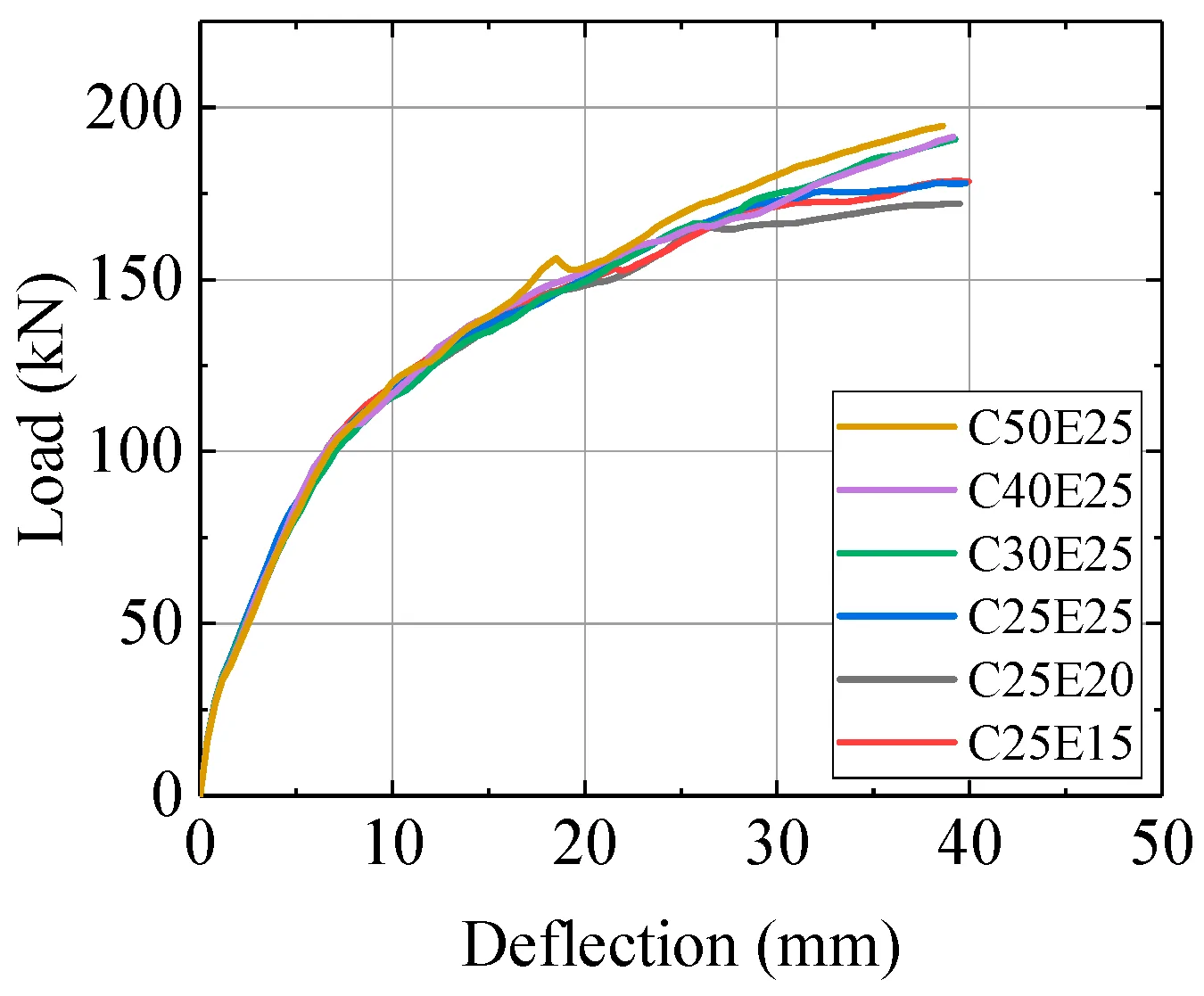
Load–deflection responses of specimen B-S10G5-E45 with different mesh configurations.

**Figure 4:**
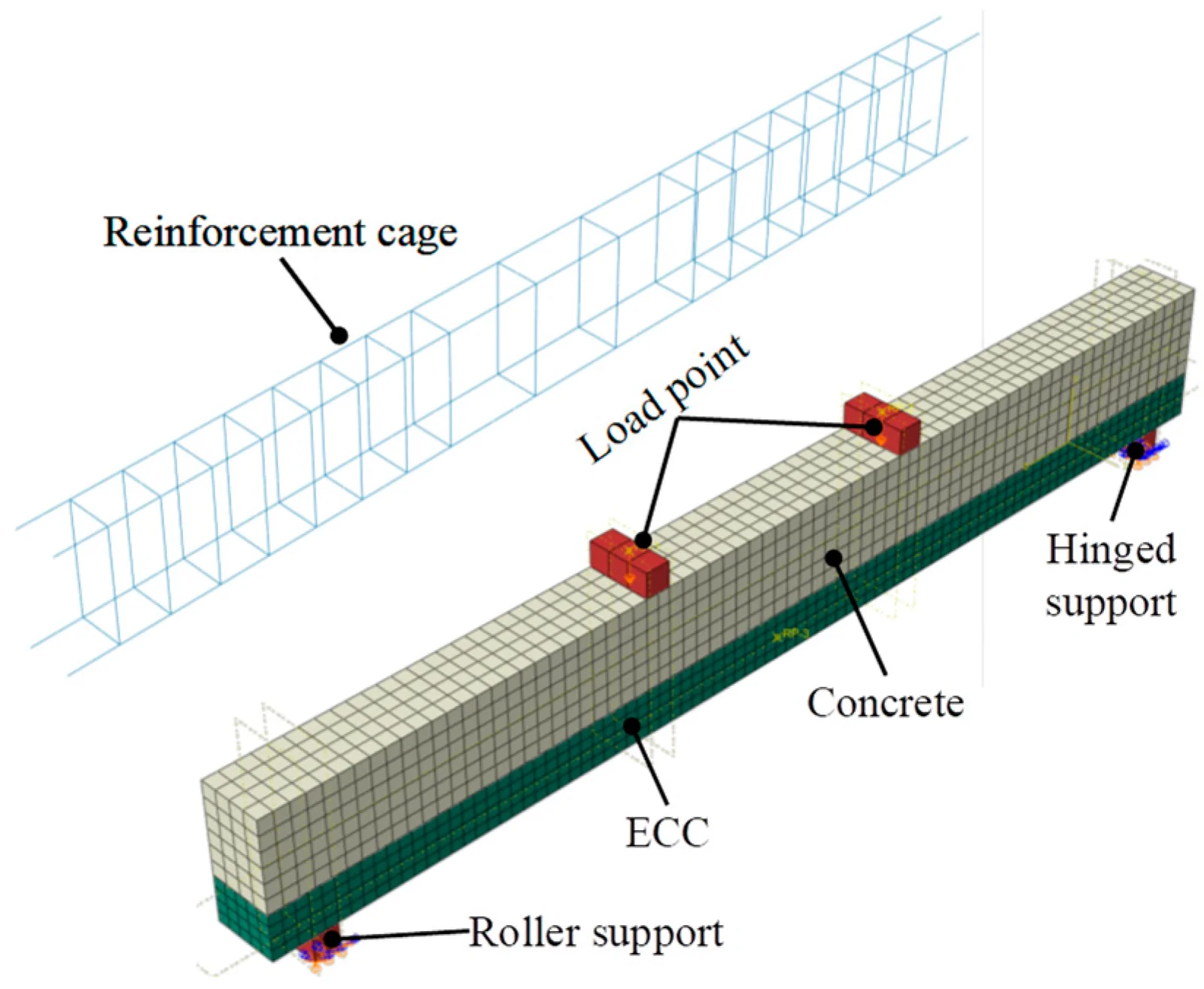
Finite element model and mesh generation.

As shown in Figure 3, the load–deflection responses obtained using different mesh configurations were generally similar during most of the loading process. The C40E25 and C30E25 models produced nearly identical responses, while some differences appeared near the final failure stage when the concrete mesh size was reduced to 25 mm. With the concrete mesh fixed at 25 mm, further refinement of the ECC mesh from 25 mm to 15 mm resulted in only minor changes in the global load–deflection response. Therefore, element sizes of 25 mm for concrete, 20 mm for ECC, and 25 mm for reinforcement were adopted in the subsequent analyses. The finite element model and mesh configuration are shown in Figure 4.

#### 2.4.2. Interaction

Considering the satisfactory bond performance and the absence of noticeable relative slip during the experiments, a perfect bond was assumed between the longitudinal reinforcement and the surrounding concrete in the finite element model. Therefore, the bond-slip behavior was neglected, and the Embedded Region constraint was adopted to represent the interaction between reinforcement and concrete. The ECC–concrete interface was simulated using the surface-based cohesive interaction described in Section 2.3. The contact formulation was defined using small sliding, and the discretization method was set as surface-to-surface contact. In addition, the loading pads and support pads were connected to the beam using Tie constraints. The present study focuses on the global flexural response rather than local anchorage or bond failure, and no obvious reinforcement pull-out or significant relative slip was observed in the corresponding experiments. Nevertheless, neglecting local bond slip may affect the predicted crack spacing and local strain distribution, and this simplification should be considered when interpreting the detailed cracking response.

#### 2.4.3. Boundary Conditions and Loading

The boundary conditions and loading configurations were consistent with the experimental setup. The two ends at the bottom of the beam were defined as a pinned support and a roller support, to simulate the simply supported flexural condition. Vertical displacement loading was applied at the two loading points through rigid loading pads and reference points. The distance between each support and its adjacent loading point was 700 mm, while the spacing between the two loading points was 600 mm. A displacement-controlled loading method was adopted, consistent with the experimental procedure.

### 2.5. Failure Criterion

This study focused on the overall flexural response of the composite beams. Therefore, the following failure criteria were adopted in the finite element analysis: (1) the compressive strain of concrete at the top compression zone reached 0.0035, indicating that failure is governed by concrete crushing. (2) The strain of the SFCB reached its ultimate fracture strain, indicating that failure is dominated by SFCB rupture. (3) The stirrups in the shear span reached the yield state, indicating shear failure caused by insufficient shear resistance.

## 3. Model Validation

### 3.1. Failure Mode

Overall, the numerical model reasonably reproduced the tensile strain-localization regions, compressive damage locations, and final failure characteristics of the composite beams subjected to four-point bending. In the experiments, all specimens exhibited a flexural-dominated failure mode. For the steel-reinforced and SFCB-reinforced composite beams, failure was characterized by concrete crushing in the compression zone after yielding of the bottom longitudinal reinforcement. In contrast, the GFRP-reinforced composite beams failed due to concrete crushing in the compression zone without rupture of the longitudinal reinforcement. The failure modes predicted by the finite element model were consistent with the experimental observations.

**Figure 5:**
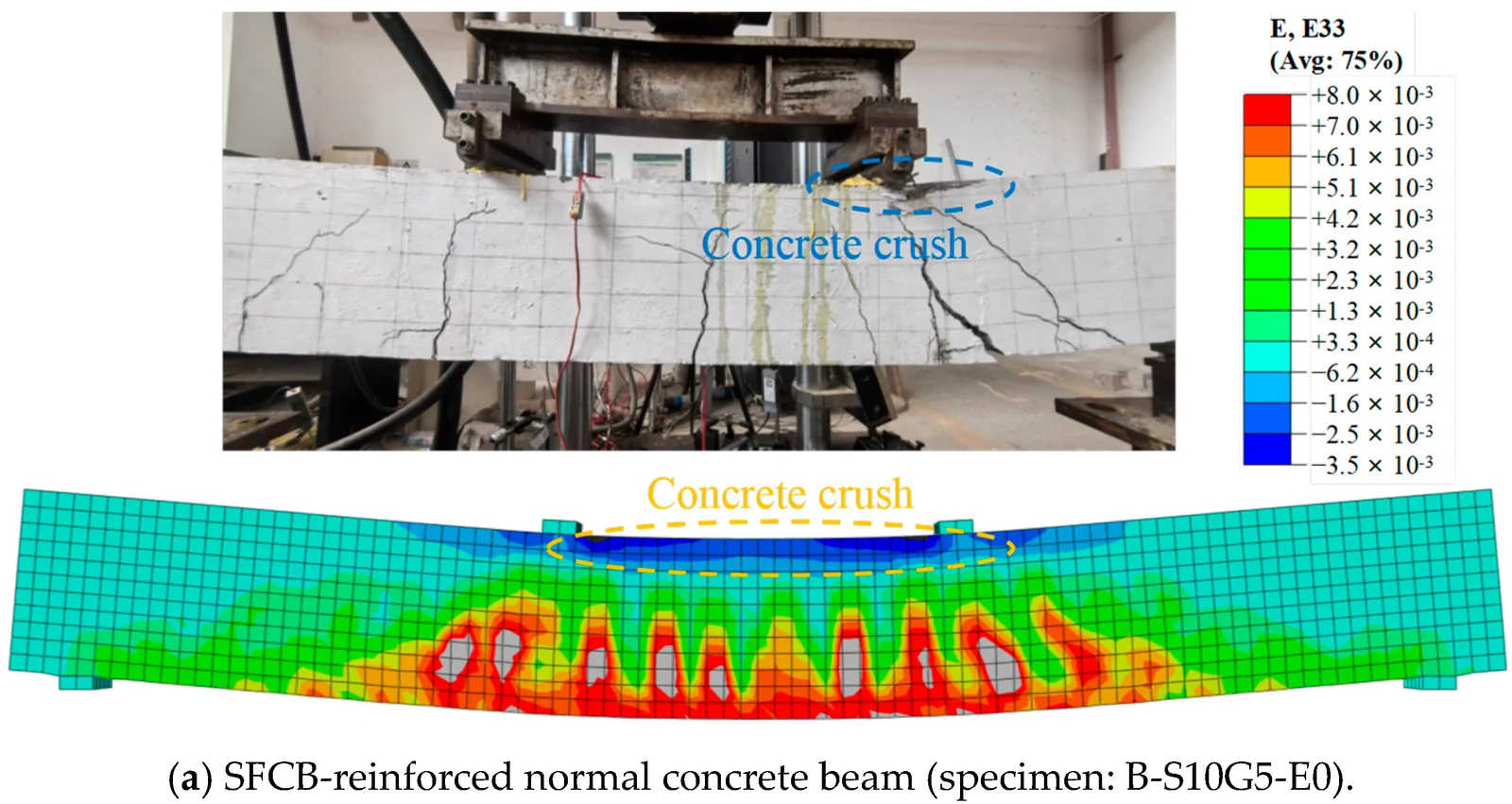

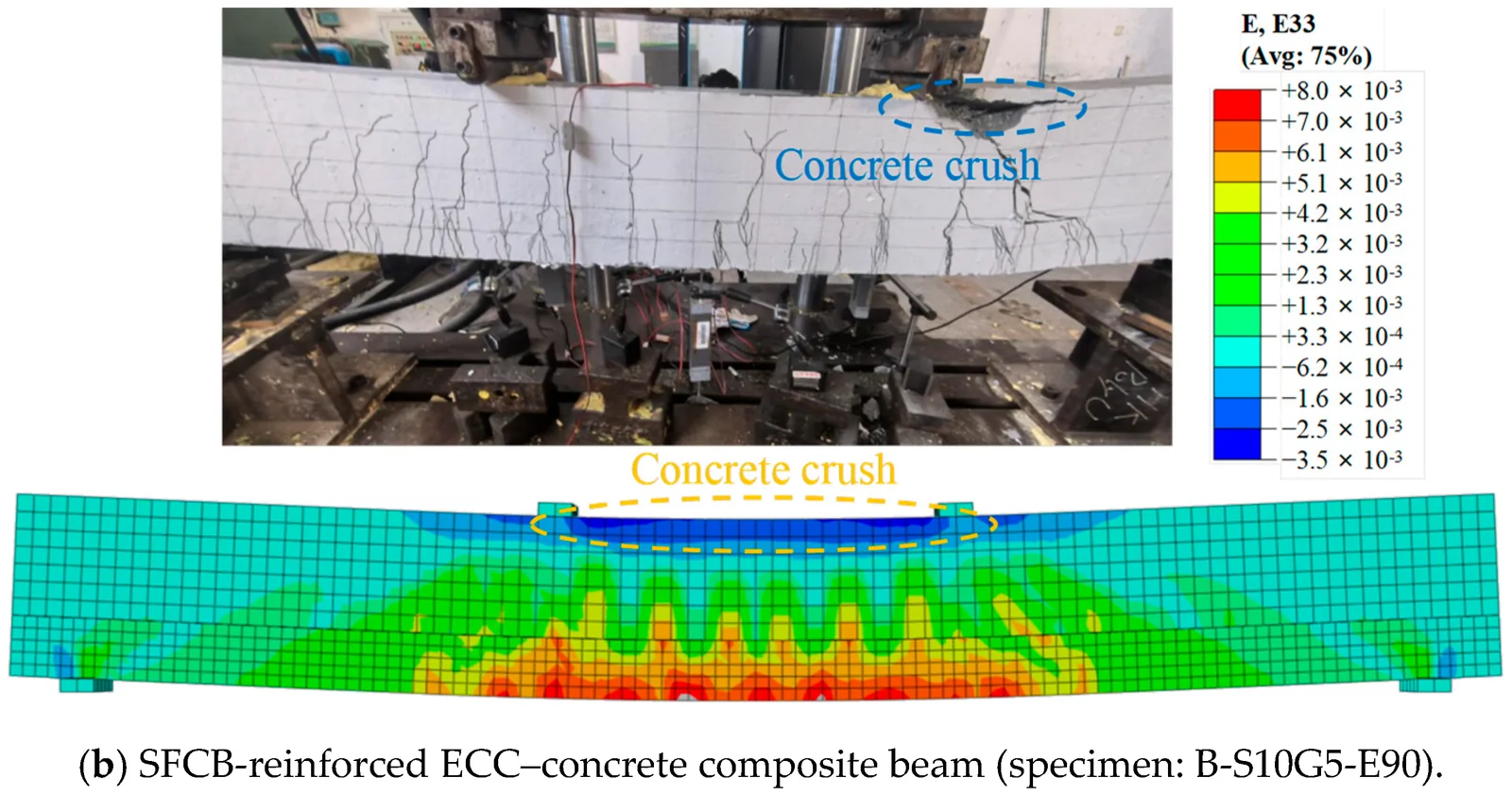
Comparison of failure modes between experimental and finite element simulations.

Typical comparisons between the finite element predictions and experimental failure patterns are presented in Figure 5. Based on the strain distribution in the tensile region, the finite element analysis showed that the main tensile strain-localization regions were concentrated within the pure bending region and near the loading points, generally consistent with the experimentally observed cracking regions [39]. The comparison results showed that the introduction of the ECC layer reduced the high-strain regions at the bottom tensile zone and resulted in a more uniform strain distribution, indicating that ECC could mitigate localized crack development. This finding agrees well with the experimental observation that ECC replacement increased the number of fine cracks and restrained the propagation of dominant cracks. It should be noted that the tensile strain and damage fields in the continuum CDP model do not correspond one-to-one with individual physical cracks. Therefore, the comparison focuses on the overall cracking regions and strain-localization tendencies.

### 3.2. Load–Deflection Curves

**Figure 6:**
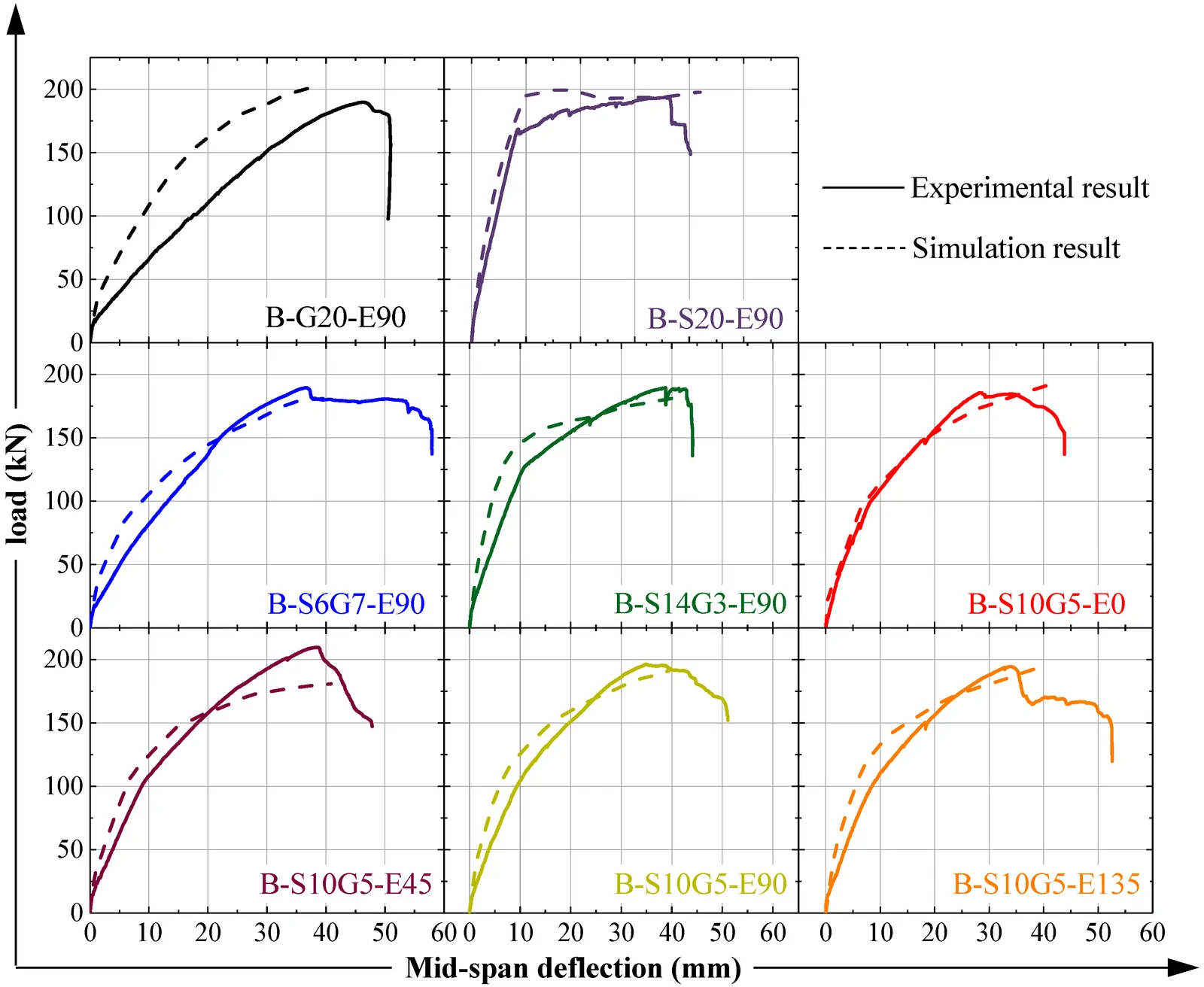
Comparison of load–deflection curves between experimental and finite element simulations.

The load–deflection curves of all specimens were obtained based on the load applied at the loading points and the mid-span deflection extracted from the finite element analysis, and were compared with the experimental results, as shown in Table 3 and Table 4 and Figure 6. Quantitative comparisons indicate that the finite element model provides relatively accurate predictions of the yielding and ultimate responses, whereas larger discrepancies are observed at the cracking stage. The mean absolute errors of the yielding load and ultimate load are approximately 3.26% and 1.34%, respectively. In contrast, the cracking load is generally overestimated, and relatively large deviations are also observed in the corresponding cracking and yielding deflections. These discrepancies are mainly associated with the sensitivity of crack initiation and early-stage deformation to the tensile constitutive description of the cementitious materials and the idealizations inherent in the continuum finite element model. Overall, the model reasonably reproduces the global flexural response and ultimate load-carrying behavior of the investigated beams, while the prediction of crack initiation remains less accurate.

## 4. Parameter Analysis

**Figure 7:**
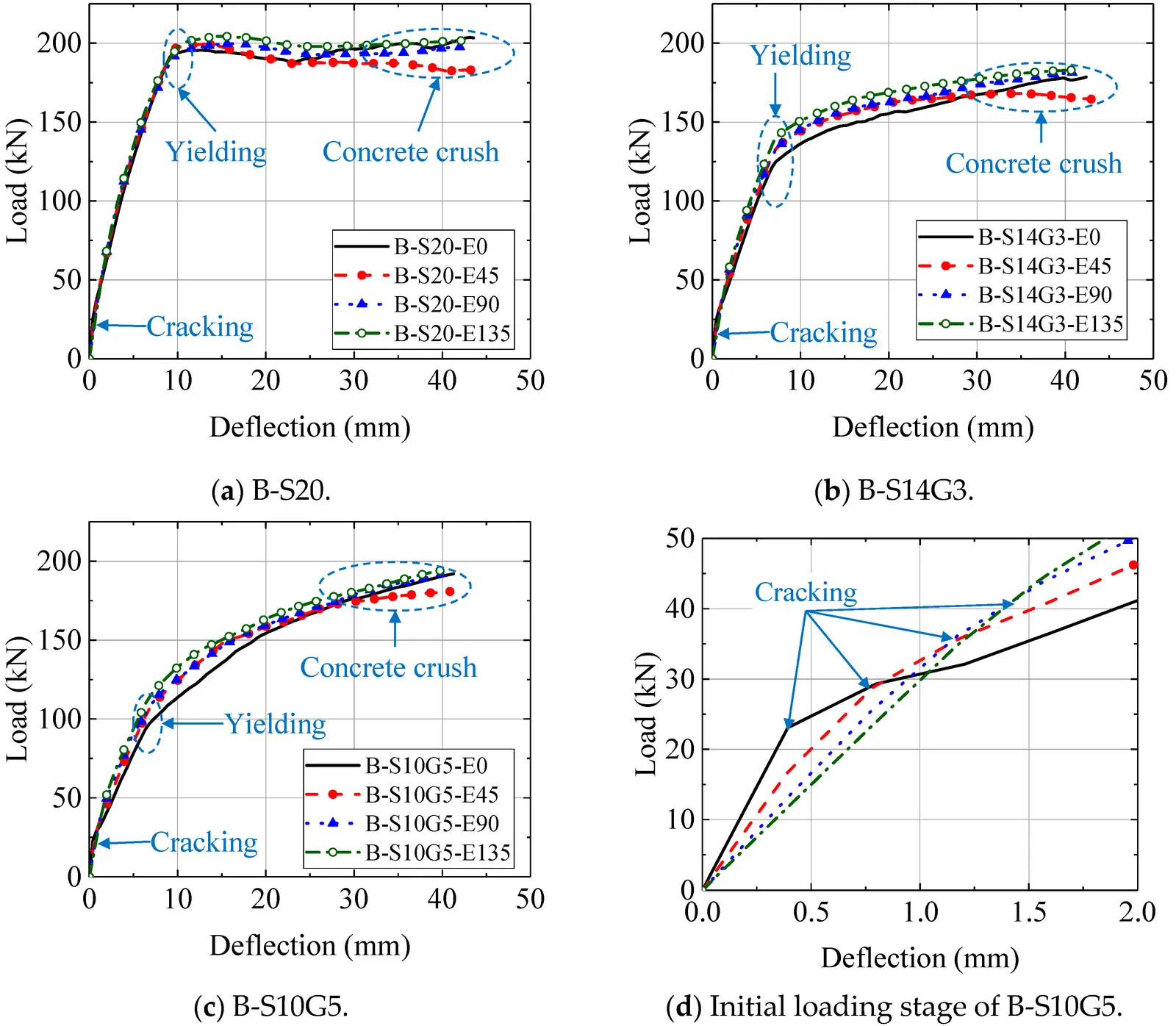

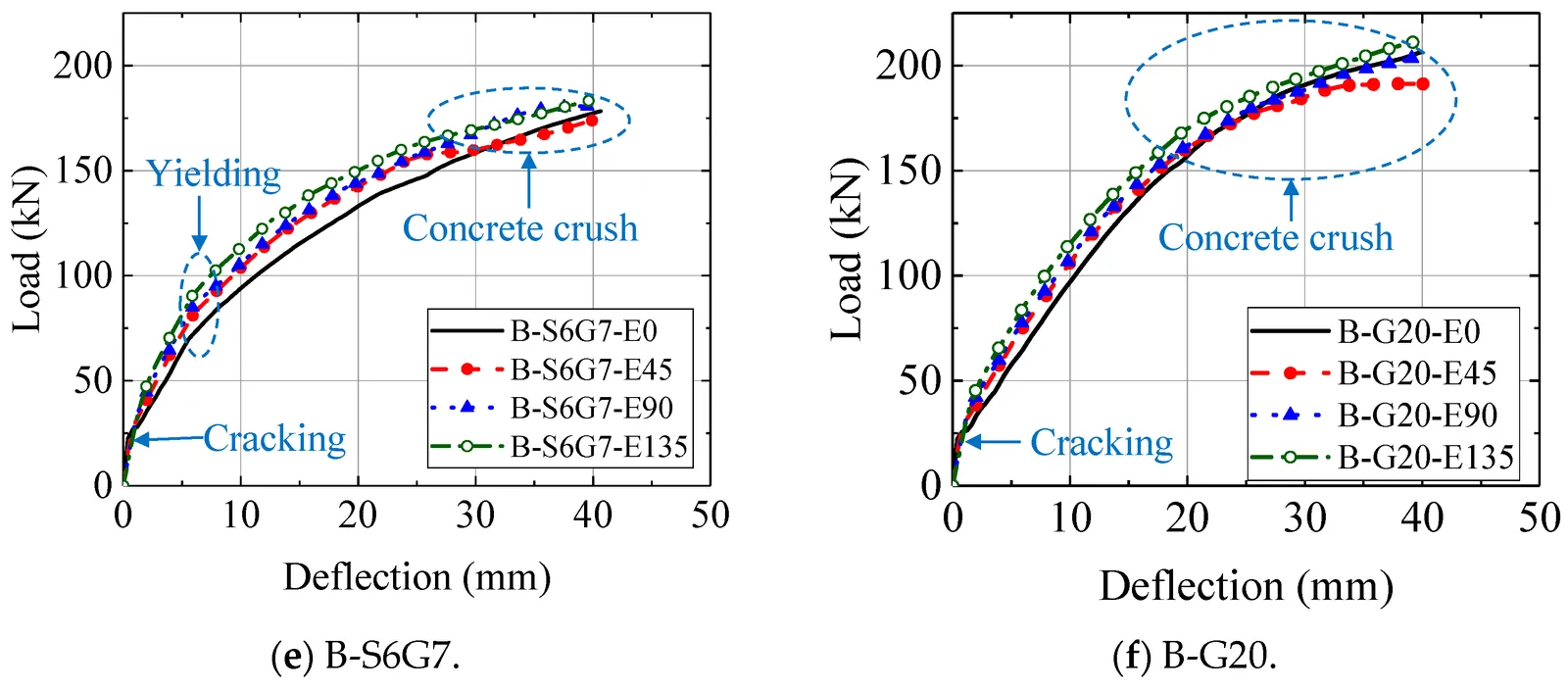
Load–deflection curves by finite element simulations.

Based on the validated finite element model, further parametric analyses were conducted to investigate the effects of ECC replacement height and bottom longitudinal reinforcement type on the flexural performance of GFRP–steel composite bar-reinforced ECC–concrete composite beams. Four ECC replacement heights (*h_E_* = 0, 45, 90, and 135 mm) were considered, and composite beams reinforced with SFCBs with different steel ratios, pure GFRP bars, and steel bars were included in the parametric study. Figure 7 presents the load–deflection responses of the composite beams with different ECC replacement heights. Overall, increasing the ECC replacement height resulted in a certain improvement in the load–deflection responses of different types of composite beams. However, the differences among specimens with different ECC thicknesses were relatively limited, indicating that the ECC replacement height was not the dominant factor governing the overall load-carrying performance of the composite beams. In contrast, the type of longitudinal reinforcement and steel ratio had a more pronounced influence on the flexural behavior, which is consistent with previous experimental observations [39].

The enlarged load–deflection curves of beam B-S10G5 in Figure 7d show that all specimens remained uncracked during the initial loading stage, and the tensile force was jointly resisted by the concrete and longitudinal reinforcement. Since the elastic modulus of ECC was considerably lower than that of normal concrete, increasing the ECC replacement height resulted in a noticeable reduction in the initial stiffness of the specimens. After cracking of the concrete, the tensile contribution of normal concrete gradually diminished, and the applied load was progressively transferred to the longitudinal reinforcement. Compared with specimens without ECC, the beams incorporating an ECC layer exhibited a slower stiffness degradation during crack formation due to the multiple-cracking behavior and fiber-bridging effect of ECC. This tendency became more evident with increasing ECC replacement height. However, because the tensile strength of ECC was only moderately higher than that of normal concrete, its direct contribution to the ultimate flexural capacity of the cross-section was limited. Therefore, increasing the ECC replacement height did not result in a significant increase in the load-carrying capacity of the composite beams.

**Figure 8:**
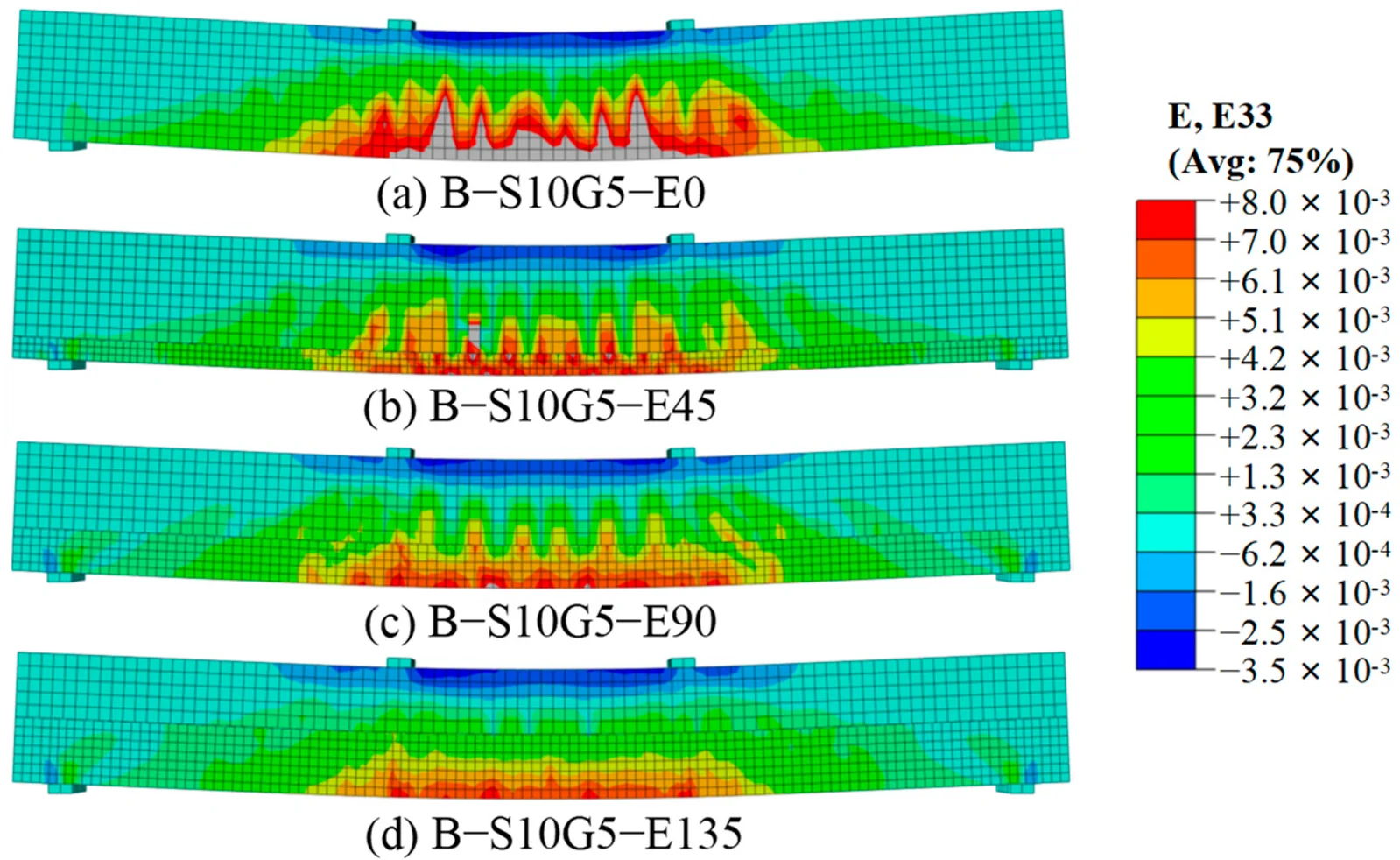
Strain cloud map by finite element simulations.

Figure 8 presents the strain distribution contours of the B-S10G5 composite beams with different ECC replacement heights. It can be observed that increasing the ECC replacement height gradually reduced the high-strain concentration in the bottom tensile region, resulting in a more uniform strain distribution. This behavior is consistent with the gradual redistribution of tensile stresses after cracking. For the specimens without an ECC layer, localized high-strain regions were prone to develop near the dominant cracks after tensile cracking, leading to concentrated damage around the main crack. In contrast, the introduction of an ECC layer restricted crack propagation and alleviated local stress concentration due to the strain-hardening behavior and fiber-bridging effect of ECC. Consequently, the tensile region could accommodate deformation through the formation of multiple fine cracks.

Therefore, the primary contribution of ECC lies in modifying the crack development pattern and improving deformation compatibility rather than significantly increasing the overall load-carrying capacity. With increasing ECC replacement height, the participating region of ECC in the tensile zone was enlarged, further improving the post-cracking strain distribution. This observation agrees with the smoother post-cracking load–deflection responses shown in Figure 8, indicating that ECC enhances the crack resistance and deformation performance of composite beams mainly by regulating the damage evolution process in the tensile zone.

**Figure 9:**
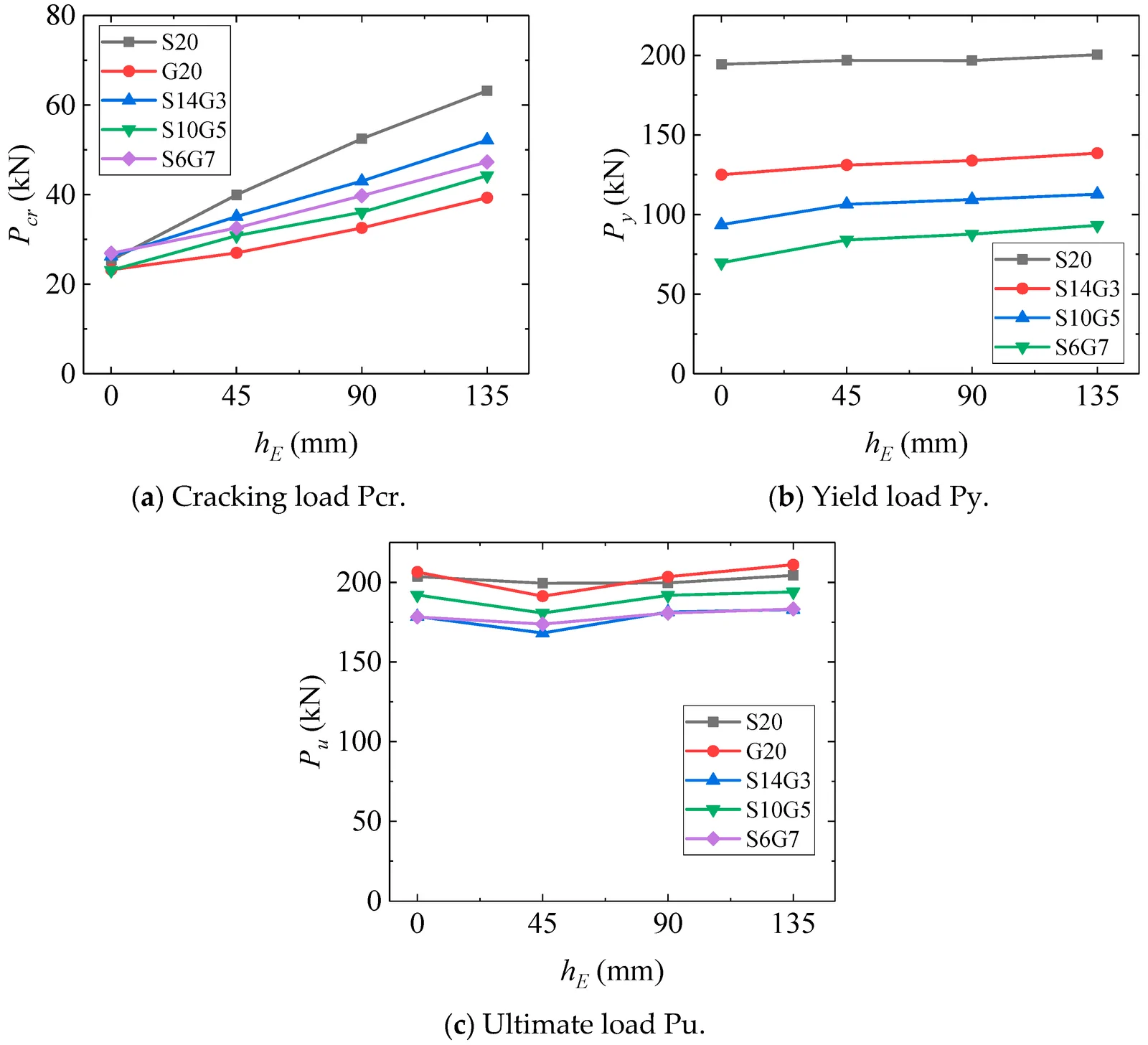
Variation in characteristic loads of composite beams.

To further evaluate the influence of ECC replacement height on the mechanical response of composite beams at different loading stages, Figure 9 presents the variations in cracking load (*P_cr_*), yielding load (*P_y_*), and ultimate load (*P_u_*) of the composite beams with different ECC replacement heights. As shown in Figure 9a, the ECC replacement height had the most pronounced influence on the cracking load. With increasing ECC replacement height, the cracking loads of all composite beams exhibited an increasing trend, indicating that the ECC layer effectively improved the cracking resistance of the tensile zone. For example, the Pcr of specimen S10G5 increased from 23.08 kN without ECC to 44.26 kN at *h_E_* = 135 mm, corresponding to an increase of approximately 91.8%. For specimen S20, the cracking load increased from 25.37 kN to 63.21 kN, representing an improvement of 149.1%. This behavior can be attributed to the relatively high tensile strength and strain-hardening characteristics of ECC, which enable the ECC layer to sustain part of the tensile stress before cracking of normal concrete, thereby delaying the initiation of cracks.

It was also observed that the effectiveness of ECC in improving the cracking load was related to the type of longitudinal reinforcement. Under the same ECC replacement height, SFCB-reinforced beams with higher steel ratios and steel-reinforced beams generally exhibited higher cracking loads. For instance, when *h_E_* = 90 mm, the cracking loads of S20 and S14G3 were 52.50 kN and 43.02 kN, respectively. This indicates that the reinforcement stiffness and tensile-zone material properties jointly affect the stress state during the initial cracking stage.

Figure 9b illustrates the effect of ECC replacement height on the yielding load (*P_y_*). Compared with the cracking load, the influence of ECC replacement height on the yielding load was considerably reduced. Taking specimen S10G5 as an example, when hE increased from 0 mm to 135 mm, *P_y_* increased from 93.58 kN to 112.85 kN, corresponding to an increase of only approximately 20.6%. This indicates that the longitudinal reinforcement gradually became the primary load-carrying component after entering the yielding stage, limiting the contribution of ECC to the yielding capacity. The differences among various reinforcement types further demonstrate that the steel content of SFCBs plays a dominant role in determining the yielding behavior.

As shown in Figure 9c, the ECC replacement height had a relatively limited influence on the ultimate load (*P_u_*). Under different ECC replacement heights, only minor variations in the ultimate loads were observed for all types of composite beams. For instance, the *P_u_* values of specimen S10G5 ranged from 180.73 kN to 194.00 kN, while the ultimate loads of S20 specimens remained nearly unchanged at approximately 200 kN under different ECC replacement heights. These results indicate that, at the ultimate state, the load-carrying capacity of the composite beams was mainly governed by the reinforcement properties and the crushing behavior of the concrete in the compression zone. Since the ECC layer was located in the tensile region, its contribution to the improvement of the ultimate load was limited. Moreover, the compression-zone concrete reached its crushing strain before the tensile strain of ECC reached its peak value of 0.055 in all simulated specimens. Therefore, the simplified compressive constitutive treatment of ECC is expected to have only a limited influence on the predicted ultimate load for the beam configurations considered in this study. From a practical perspective, increasing the ECC replacement height mainly improves cracking resistance, whereas the additional improvement in yielding and ultimate loads becomes relatively limited. Therefore, for the beam configuration investigated in this study, an ECC replacement height of 45 mm (*h_E_*/*h* = 16.7%) may provide a preliminary balance between ECC consumption and flexural performance. This value should not be regarded as a universal optimum, and further cost and life-cycle assessments are required for general engineering application.

## 5. Conclusions

A three-dimensional nonlinear finite element model of SFCB-reinforced ECC–concrete composite beams was developed using ABAQUS. The effects of ECC replacement height and bottom longitudinal reinforcement type on the flexural performance of composite beams were investigated. The main conclusions are summarized as follows:

(1) The developed finite element model can reasonably predict the load–deflection responses, crack development, and failure process of the composite beams. The composite beams mainly exhibited flexural-dominated failure modes, involving crack propagation in the tensile zone, progressive transfer of tensile forces to the longitudinal reinforcement, and damage evolution of concrete in the compression zone.
(2) The type of longitudinal reinforcement had a significant influence on the flexural behavior of composite beams. Different reinforcement types resulted in distinct load-carrying capacities, stiffness evolution characteristics, and deformation responses. The steel core in SFCBs effectively improved the insufficient ductility of GFRP reinforcement.
(3) The ECC replacement height mainly affected the cracking resistance and crack development behavior of the composite beams. Increasing the ECC replacement height increased the cracking load, improved the strain distribution in the tensile zone, and mitigated localized crack development. However, its contribution to the yielding load and ultimate load was relatively limited.
(4) ECC and SFCBs exhibited a synergistic effect on the composite beams. ECC primarily contributed to crack control and damage regulation, while SFCBs provided load-carrying capacity and ductility. An appropriate combination of ECC replacement height and SFCB parameters can improve the overall crack resistance, load-carrying performance, and deformation performance of composite beams.

## Figures and Tables

**Table 1:** Parameters of the concrete plastic damage model.

Angle of Dilatancy	Flow Potential Offset	Ultimate Strength Ratio of Biaxial Compression to Uniaxial Compression	Invariant Stress Ratio	Viscosity Coefficient
30	0.1	1.16	0.667	0.0005

**Table 2:** Cohesive contact parameters for ECC–concrete interface.

Parameter	Value
Normal initial stiffness *K_nn_*	33,000 *N*/mm^3^
Shear initial stiffness *K_ss_* = *K_tt_*	33,000 *N*/mm^3^
Normal damage initiation stress *τ_n_*	8.59 MPa
Shear damage initiation stress *τ_s_* = *τ_t_*	12.65 MPa
Fracture energy *G_f_*	0.85 *N*/mm
Viscosity coefficient *η*	1 × 10^−5^

**Table 3:** Quantitative comparison between experimental and FE results.

Specimens	Experimental Value			Calculated Values by FEM					
	*P_cr_* (kN)	*P_y_* (kN)	*P_u_* (kN)	*P_cr_* kN)	Error (%)	*P_y_* (kN)	Error (%)	*P_u_* (kN)	Error (%)
B-S10G5-E0	17.01	102.09	185.48	23.08	35.68	93.58	−8.34	192.0128	3.52
B-S10G5-E45	22.17	104.31	209.34	30.83	39.06	106.46	2.06	180.7342	−13.66
B-S10G5-E90	22.63	104.94	196.23	36.07	59.39	109.46	4.31	191.815	−2.25
B-S10G5-E135	21.8	105.35	194.38	44.26	103.03	112.85	7.12	194.0042	−0.19
B-S20-E90	24.25	168.28	193.06	52.5	116.49	196.66	16.86	199.6318	3.40
B-S14G3-E90	23.19	126.63	189.34	43.02	85.51	133.87	5.72	181.4076	−4.19
B-S6G7-E90	21.95	92.15	189.47	39.74	81.05	87.64	−4.89	180.6716	−4.64
B-G20-E90	22.21	/	189.67	32.57	46.65	/	/	203.482	7.28

Note: *P_cr_*, *P_y_* and *P_u_* are the load-carrying capacities at cracking, yielding, and ultimate states, respectively.

**Table 4:** Quantitative comparison between experimental and FE results.

Specimens	Experimental Value			Calculated Values by FEM					
	*Δ_cr_* (kN)	*Δ_y_* (kN)	*Δ_u_* (kN)	*Δ_cr_* kN)	Error (%)	*Δ_y_* (kN)	Error (%)	*Δ_u_* (kN)	Error (%)
B-S10G5-E0	0.93	8.71	28.4	0.39248	−57.80	5.96204	−31.55	41.2588	45.28
B-S10G5-E45	0.95	9.32	38.69	0.78422	−17.45	6.75778	−27.49	40.8726	5.64
B-S10G5-E90	1.28	10.15	35.06	1.16875	−8.69	7.06205	−30.42	39.8291	13.60
B-S10G5-E135	1.07	9.39	34.34	1.16564	8.94	6.25984	−33.34	39.7395	15.72
B-S20-E90	0.63	8.54	35.46	1.16606	85.09	10.407	21.86	41.9879	18.41
B-S14G3-E90	1.05	10.8	36.67	1.16741	11.18	7.43384	−31.17	40.7956	11.25
B-S6G7-E90	1.45	11.81	38.74	1.17007	−19.31	5.88731	−50.15	39.5206	2.01
B-G20-E90	1.59	/	46.59	0.77941	−50.98	/	/	39.1163	−16.04

Note: *Δ_cr_*, *Δ_y_* and *Δ_u_* are the mid-span deflection at cracking, yielding, and ultimate states, respectively.

## Data Availability

The original contributions presented in this study are included in the article. Further inquiries can be directed to the corresponding author.
